# Antiviral Activities of Sulfated Polysaccharides Isolated from *Sphaerococcus coronopifolius* (*Rhodophytha*, *Gigartinales*) and *Boergeseniella thuyoides* (*Rhodophyta*, *Ceramiales*)

**DOI:** 10.3390/md9071187

**Published:** 2011-07-06

**Authors:** Rhimou Bouhlal, Camille Haslin, Jean-Claude Chermann, Sylvia Colliec-Jouault, Corinne Sinquin, Gaelle Simon, Stephane Cerantola, Hassane Riadi, Nathalie Bourgougnon

**Affiliations:** 1 Laboratoire de Biotechnologie et Chimie Marines (LBCM), Centre de recherche Yves Coppens, Université européenne de Bretagne (UEB), Université de Bretagne-Sud. Vannes, France; 2 Laboratoire de Diversité et Conservation de Systèmes Biologiques (LDICOSYB), Faculté de Science, Université Abdelmalek Essaâdi, Tétouan, Maroc. B.P. 2121, 93003, Morocco; E-Mail: hassaner@hotmail.com; 3 URRMA R&D, Centre de vie agora, bâtiment C, B.P. 1055, Z.I. des paluds, 13781 Aubagne Cedex, France; E-Mails: cherma@urrma.eu (C.H.); haslin@urrma.eu (J.-C.C.); 4 Laboratoire Biotechnologies et Molécules Marines, IFREMER, B.P. 21105 44311 Nantes, France; E-Mails: sylvia.colliec.jouault@ifremer.fr (S.C.-J.); corinne.sinquin@ifremer.fr (C.S.); 5 Université européenne de Bretagne, Université de Bretagne Occidentale, Service Commun de Résonance Magnétique Nucléaire, UFR Sciences et Techniques, Avenue Le Gorgeu, CS 93837 29238 Brest Cedex 3, France; E-Mails: gaelle.simon@univ-brest.fr (G.S.); stephane.cerantola@univ-brest.fr (S.C.)

**Keywords:** *Herpes simplex* virus, human immunodeficiency virus, antiviral activity, sulfated polysaccharide, *S. coronopifolius*, *B. thuyoides*, chemical composition

## Abstract

Water-soluble sulfated polysaccharides isolated from two red algae *Sphaerococcus coronopifolius* (*Gigartinales*, *Sphaerococcaceae*) and *Boergeseniella thuyoides* (Ceramiales, Rhodomelaceae) collected on the coast of Morocco inhibited *in vitro* replication of the Human Immunodeficiency Virus (HIV) at 12.5 μg/mL. In addition, polysaccharides were capable of inhibiting the *in vitro* replication of *Herpes simplex* virus type 1 (HSV-1) on Vero cells values of EC_50_ of 4.1 and 17.2 μg/mL, respectively. The adsorption step of HSV-1 to the host cell seems to be the specific target for polysaccharide action. While for HIV-1, these results suggest a direct inhibitory effect on HIV-1 replication by controlling the appearance of the new generations of virus and potential virucidal effect. The polysaccharides from *S. coronopifolius* (PSC) and *B. thuyoides* (PBT) were composed of galactose, 3,6-anhydrogalactose, uronics acids, sulfate in ratios of 33.1, 11.0, 7.7 and 24.0% (w/w) and 25.4, 16.0, 3.2, 7.6% (w/w), respectively.

## 1. Introduction

Marine seaweeds have proven to be exceptionally rich sources for biologically active metabolites [1–5]. Polysaccharides extracted from Rhodophyta have been shown to exhibit antiviral activity against a wide spectrum of viruses including important human pathogenic agents such as Human Immunodeficiency virus (HIV), *Herpes simplex* virus (HSV), *Vesicular stomatitis* virus (VSV), Cytomegalovirus **(**CMV) [6–13]. With known biochemical and cellular actions, polysaccharides offer a number of promising features.

Polysaccharides from red seaweeds include different sulfated galactans, sulfated rhamnans or mannans, carrageenans and agars [6,9,11,13,14]. The predominant types of polysaccharide from red algae are galactans with a backbone of alternating 3-linked β-d-galactopyranosyl and 4-linked α-galactopyranosyl units: agar-type polysaccharide if the 4-linked α-galactopyranosyl units are in the l-configuration, and a carrageenan-type polysaccharide, if the 4-linked α-galactopyranosyl units are in the d-configuration [15]. Agars are typically low in sulfate ester substitution, but those from numerous sources are rich in methyl ether or pyruvate acetal substitution. Conversely, carrageenans are comparatively rich in sulfate ester substitution but poor in methyl ether substitution, and rarely contain significant levels of pyruvate acetal substitution [16]. Their modes of action have been attributed to the blockage of some early stages of the virus replication cycle [17,18].

The Moroccan Coast is particularly rich in algal biodiversity and constitutes a reserve of species of considerable economic, social and ecologic potential. Except studies of Abourriche *et al*. [19], Etahiri *et al*. [20], Moujahidi *et al*. [21], and Souhaili *et al*. [22] concerning antibacterial activities, nothing is known about the antiviral activities of red seaweed extracts collected on the coast of Morocco.

The present study lead to elucidating the chemical composition and antiviral activities against two enveloped viruses HSV-1 and HIV of water-soluble polysaccharides extracted from *Sphaerococcus coronopifolius* (*Gigartinales*, *Sphaerococcaceae*) and *Boergeseniella thuyoides* (*Ceramiales Rhodomelaceae*) and the first approach *in vitro* of time of action.

## 2. Results and Discussion

### 2.1. Chemical Composition from the Polysaccharides

Extraction yields from *Sphaerococcus coronopifolius* and *Boergeseniella thuyoides* were 25.5% and 17.8% respectively.

The chemical compositions of water-soluble polysaccharides are shown in Table 1.

The polysaccharide from *S. coronopifolius* contains more galactose, uronic acid and sulfates than that of *B. thuyoides*. The latter contains more 3,6-anhydrogalactose and proteins.

The ability to synthesize acid polysaccharides is the most interesting property of red seaweeds. Several species produce complex heteropolysaccharides containing uronic acids together with neutral or sulfated monosaccharides or galactans [23,24]. Red algal galactans constitute a spectrum of polysaccharides encompassing a variety of non-glycosyl substitutions (methoxyl, pyruvate, sulfate groups, other sugar residues (galactose, xylose) or uronic acids. In our study, the polysaccharide of *S. coronopifolius* and *B. thuyoides* respectively contain 7.7% and 3.2% of uronic acids. Aqueous extraction of gametophytic *Schizymenia binderi* (*Gigartinales*, *Schizymeniaceae*) afforded a polysaccharide composed of galactose and sulfate groups in a molar ratio of 1.0/0.89 together with uronic acids (6.8 wt%) and minor amounts of other neutral sugars [9]. Chemical and spectroscopic methods showed that the water-soluble polysaccharide from *Schizymenia dubyi* (*Gigartinales*, *Schizymeniaceae*) is a sulfated heteropolysaccharide with uronic acid. Galactose, 3,6-anhydrogalactose and sulfate (molar ratio): 1/0.75/1.3/33.7% (dry weight) of uronic acids [25]. The ontogenesis of the Mediterranean invasive species *Asparagopsis armata* (*Bonnemaisoniales*, *Bonnemaisoniaceae*) is an alternation of morphologically dissimilar gametic, carposporic, and tetrasporic reproductive phases which produced complex sulfated galactans composed of galactose: anhydrogalactose: sulfates in molar ratios of 1/0.01/1.23, 1/0.04/0.47 and 1/0.01/1.13, respectively [26]. These peculiar polymers of 1,3- and 1,4-linked galactose units, containing uronic acids (3.5–15.9% dry weight) and in which d-galactose content (77.6–92.6% of total galactose) exceeds l-galactose content, do not agree with ideal carrageenan type polysaccharides.

Chemical composition of agar isolated from *Gelidium purpurascens* (*Gelidiales*, *Gelidiaceae*), shows the presence of glucuronic acid [27]. Chemical analysis of *Gracilaria verrucosa* (*Gracilariales*, *Gracilariaceae*) showed that these fractions contained increasing proportions of galactose, uronic acid, and sulfate residues and decreasing proportions of 6-*O*-methylgalatose and 3,6-anhydrogalactose residues [28].

### 2.2. Determination of the Molecular Weight of Polysaccharides

The weight average molecular weight (Mw) of polysaccharides is about 308,700 Da and 360,300 Da for *S. coronopifolius* and *B. thuyoides* respectively. The number average molecular weight (Mn) is about 52,400 Da and 171,500 Da with an index of polydispersity (Mw/Mn) of 2.101 and 5.891 for *S. coronopifolius* and *B. thuyoides* respectively. The polysaccharide from *S. coronopifolius* is more homogeneous than that of *B. thuyoides*.

### 2.3. Spectroscopic Analysis

The native polysaccharides contained strong absorption bands between 1250–1370 cm^−1^ indicative of S=O stretching vibration of sulfate groups [29–33]. The diagnostic regional 1609–1420 is characteristic of uronic acids [34–36].

#### Fourier Transformed Infra Red (FTIR) Spectroscopy Analysis

**Figure 1:**
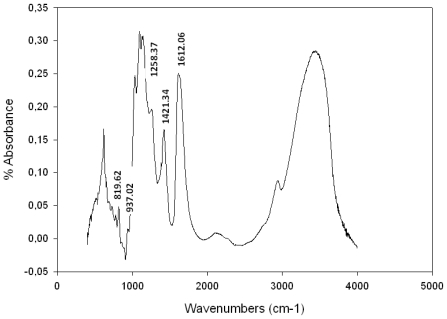
FTIR spectrum of polysaccharide of *S. coronopifolius*.

The signal at 937 cm^−1^ (Figure 1) was attributable to 3,6-anhydrogalactose residues [30]. The diagnostic region of the FTIR spectra between 815 and 830 cm^−1^ of native galactan contained one major absorption band at 819 cm^−1^, is effectively well known for λ-carrageenans [10,11,31,37,38], because of its diagnostic sulfate ester substitution at O-6 of 4-3-linked galactose residues [31,37].

The chemical composition and structural features of *S. coronopifolius* water-soluble polysaccharide has never been described in the literature. The galactan presented the highest percentage of sulfated content (24%). Figure 1 shows bands at 819 cm^−1^ characteristic of λ-carrageenan. The presence of pyruvated groups was observed [10,11,31,37,38].

**Figure 2:**
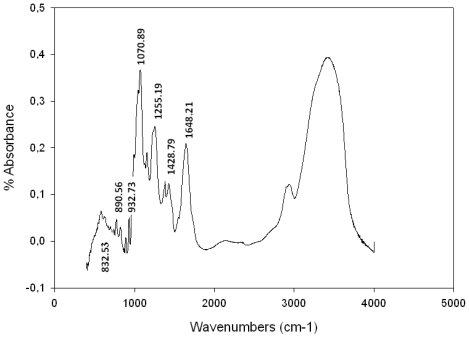
FTIR spectrum of polysaccharide of *B. thuyoides*.

The diagnostic region (940–800 cm^−1^) showed a band at 932 cm^−1^ (Figure 2) indicating the presence of 3,6-anhydrogalactose [11,30,32–34,37–41]. Additionally a peak was observed at 890 cm^−1^ that is characteristic of agar [42,43]. The bands around 1070 cm^−1^ and 930 cm^−1^ are associated with the presence of C3-*O*-C6 bridge of the anhydrogalactose residue [37].

The chemical composition of *Boergeseniella thuyoides* (*Polysiphonia thuyoides*) water-soluble polysaccharide has never been described in the literature, although that of *P. morrowii*, *P. strictissima*, *P. abscissoides*, *P. atterima* and *P. nigrescens* have been reported by Usov *et al*. [44,45]; Miller and Furneaux [46], Miller [47] and Prado *et al*. [48] respectively. These polysaccharides are sulfated galactans of the agaran type consisting of linear chains of alternating 3-linked β-d-galactopyranosyl and 4-linked α-l-galactopyranosyl units; some of the latter also occur in the 3, 6-anhydro form. This regular backbone is usually masked by different *O*-linked groups, particularly methyl ether with sulfate ether and β-d-xylopyranosyl residues [47]. Chemical analysis of the agaran of *Polysiphonia nigrescens* (*Rhodomelaceae*, *Ceramiales*) showed that it is highly substituted on C-6 mainly with sulfate, although methyl ester and single stubs of β-d-xylose were found in minor proportions [47]. *Polysiphonia lanosa* presents 6-*O*-methyl-d-galactose, d-galactose 6-sulfate, 6-*O*-methyl-d-galactose 4-sulfate; the 4-linked units include l-galactose 6-sulfate, 2-*O*-methyl-l-galactose 6-sulfate and 3,6-anhydr-l-galactose [49]. The chemical and spectral data from sulfated galactan of *B. thuyoides* seem to confirm the membership of polysaccharide to agaran family. Signal of bands at 1255, 1070, 932, 890, 740, 716 cm^−1^ are characteristic bands of agarocolloids [42,43]. Moreover, the galactan showed a low sulfated content (7.6%) and did not present the methoxyl and pyruvate groups.

### 2.4. Cytotoxicity and Antiviral Activity

#### 2.4.1. HSV/Vero Model

**Figure 3:**
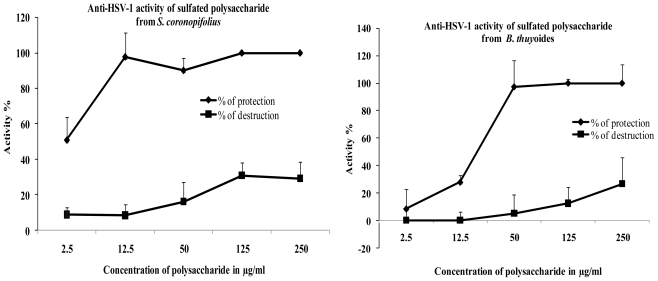
Anti-HSV-1 activities of polysaccharides at 72 h. Antiviral activity is expressed as the percentage of viable virus-infected cells (% of protection). Cytotoxic activity is observed as the percentage of viable mock-infected cells (% of destruction).

Low percentage of cell destruction was observed for the two polysaccharides on Vero cells line (CC_50_ > 250.0 μg/mL) (Figure 3). After three days of treatment, no microscopically visible alteration of normal cell morphology was observed and viability assay showed no destruction of cell layer.

When polysaccharides and the viral suspension (MOI 0.001 ID_50_/cells) are simultaneously added, 100% of cellular protection was obtained for 12.5 μg/mL of polysaccharide from *S. coronopifolius* (EC_50_ = 4.1 μg/mL). For polysaccharide extracted from *B. thuyoides*, 100% of cellular protection was obtained for 50μg/mL (EC_50_ = 17.2 μg/mL) (Figure 3). Acyclovir used as positive controls conferred total protection (100%) against HSV-1 with a low percentage of cell destruction (8%). In comparison with Acyclovir (SI: >500), the selectivity index (SI: 61 for polysaccharide extracted from *S. coronopifolius* and SI 14.5 for polysaccharide extracted from *B. thuyoides*) are low, but this study highlights the presence of an antiviral activity of marine sulfated polysaccharides.

The marine sulfated polysaccharides belonging to the Gigartinales recognized for the production of carrageenans [46,50], were shown to be potent and selective inhibitors of HIV-1 and HSV-1 replication in culture [6,13,44,51]. The sulfated galactan from *Schizymenia binderi* exhibited highly selective antiviral activity against *Herpes simplex* virus type 1 and 2, with selectivity indices >1000. This compound was shown to interfere with the initial adsorption of viruses to cells [16]. The kappa/iota/nu carrageenan from *Gymnogongrus griffithsiae* (*Phyllophphoraceae*, *Gigartinales*), exhibited antiherpetic activity with inhibitory concentration 50% (EC_50_) values in 0.5–5.6 μg/mL, as determined in a virus plaque reduction assay in Vero cells. The galactans lacked cytotoxic effects and showed a broad spectrum of antiviral activity against HSV-1 and HSV-2. No direct virus inactivation was observed after virions treatment with the galactans. The mode of action of these compounds could be mainly ascribed to an inhibitory effect on virus adsorption. Most importantly, a significant protection against a murine vaginal infection with HSV-2 was afforded by topical treatment with the sulfated galactans [52]. The carrageenan from *Callophyllus variegata* (*Kallymeniaceae*, *Gigartinales*) showed potent antiviral activity against *Herpes simplex* type 1 and 2 with values of IC_50_ ranging from 0.16 to 2.19 μg/mL, and against dengue type 2 with values of IC_50_ from 0.10 to 0.41 μg/mL. Polysaccharides from algae belonging to the Ceramiales, biosynthesized sulfated agarans [16,53]. The latter also showed antiviral activity, but less effective than carrageenan. Some agarans isolated from the red seaweed *Acanthofora spicifera* (*Rhodomelaceae*, *Ceramiales*) showed a very selective and potent antiviral activity against HSV-1 and HSV-2 with EC_50_ in the range of 0.6 and >50 μg/mL [54]. Further investigations are in progress to examine and confirm the efficiency of marine polysaccharide with higher MOI 0.01 and 0.1 ID_50_/cells.

##### First Approach to Determining the Mode of Action

###### Effect before Infection

Vero cells were not protected from HSV-1 infection when polysaccharides were only present before virus infection (Table 2). The polysaccharides did not induce a durable antiviral state in the target cells.

###### Virucidal Assay

The preincubation of the virus with polysaccharides from *S. coronopifolius* and *B. thuyoides* had a moderated protection. The values of effective concentration 50% were 53.2 and 73.9 μg/mL for *S. coronopifolius* and *B. thuyoides*, respectively.

###### Virus Adsorption Assay

With Treatment I, a moderated antiviral activity was reported. With Treatment II, a high percentage of cell protection was observed for two polysaccharides. With Treatment III, a higher percentage of protection than in Treatment II was noted with EC_50_ = 3.6 μg/mL for PSC and EC_50_ = 13.1 μg/mL for PBT. Vero cells were not protected from HSV-1 infection when polysaccharides were present before infection (Table 2).

###### Effect of Polysaccharide Addition Time

Vero cells were optimally protected (87–100%) when polysaccharide was added at 0–2 h after infection (Table 2). After 3 h post-infection, antiviral activity decreased dramatically. In comparison, the EC_50_ of acyclovir increased from 0.5 to 4.1 μg/mL between 0 and 5 h (Table 2).

In conclusion for the HSV-1 model:

Vero cells were not protected from HSV-1 infection when the polysaccharides were present only before virus infection. Sulfated polysaccharides did not adsorb to the cellular receptorse. The results also revealed that polysaccharides did not exert an important virucidal effect. Indeed, the preincubation of the virus with the polysaccharide for (2 h at 37 °C) protected Vero cells less against HSV-1. The EC_50_ obtained after two days of incubation at 37 °C were increased.

The lack of virucidal activity for the polysaccharides from *S. coronopifolius* and *B. thuyoides* is in accordance with previous studies that found most algal sulfated polysaccharides are not able to produce significant virion inactivation [9,12,13,55,56]. However, it is quite possible that sulfated polysaccharide could affect the virus-cell attachment by spreading a three-dimensional gel network over the whole of the cell surface. This structural modification would entail envelope alteration and concealing of virus-specific receptors sites. Such a mechanism prevents adsorption of the viruses to the cell surface and hence penetration and replication. Sulfated polysaccharides interact precisely with positively charged domains on these glycoproteins. This will result in a “shielding-off” of these domains and thus prevent the virus from binding to the negatively charged sulfated polysaccharide [14,18,57–59]. Heparan sulfate proteoglycans are assumed to be common cell ligands in the initial interaction of HSV-1 with host cell and external viral glycoprotein [58]. To determine the stage of HSV-1 infection affected by polysaccharide, cells were treated with the polymers at various times during the cycle of viral replication. The two polysaccharides were very effective when added at the early stages of virus growth (simultaneously with virus 0H) or when added 1 h after HSV-1 infection. Previous reports demonstrated the antiviral activity of complex sulfated polysaccharides extracted from various species of marine algae and suggested that sulfated polysaccharides interfered with the attachment of virions to host cells [6,7,9,12–14,24,54,59,60]. On the other hand, the assays of Harden *et al*. [14] and Carlucci *et al*. [55] showed that the polysaccharide from algae can have potent inactivating properties against HSV-1. Harden *et al*. [14] reported that polysaccharide from gametophyte from *Gigartina atropurpurea* and *Plocamium cartilagineum* had potent virucidal activity and were active at very low concentrations (0.2–0.4 μg/mL respectively). The λ-carrageenan from *Gigartina skottsbergii* [60] and the sulfated glucuronogalactan from *Schizymenia dubyi* [18], also have potent inactivation (EC_50_ = 0.5 μg/mL and 30 μg/mL, respectively) against HSV-1. Since polysaccharides antiviral action seemed to be efficacious by interference with early stage of the HSV-1 replication cycle, the effect on the viral adsorption was examined. The two polysaccharides protected Vero cells against HSV-1, however only if present during the first two hours after viral infection. The results confirm that sulfated polysaccharides exert their anti-HSV-1 activity mainly by interference with a very early stage of HSV-1 replication cycle (since replication was blocked when the compound was present during the virus adsorption period. Direct interference with the virus particle or a combined action on the virus and the cell on receptor sites, could account for the inhibitory effect of sulfated polysaccharide on the virus attachment to the host cells.

#### 2.4.2. HIV/MT4 Model

Water-soluble extracts from *S. coronopifolius* and *B. thuyoides* are not toxic on cells MT4, even with 300 μg/mL.

The antiviral activity of the products was tested on virus HIV-1 NDK with 2 dilutions (7.5 × 10^−4^ and 5 × 10^−4^) on cells MT4 (Tables 3 and 4). The inhibiting effect is analyzed by observing under the microscope the presence or absence of *syncitium* in the wells.

The polysaccharide of *S. coronopifolius* appeared to be active against HIV-1 replication until the seventh day for each concentration tested (Table 3). The polysaccharide of *B. thuyoides* also prevents HIV-induced *syncytium* formation but at the stronger concentration after the seventh day. However, at the lowest concentrations, *syncitia* formation was observed (Table 4). Otherwise no cytotoxicity on the MT4 cells was observed.

The marine sulfated polysaccharides were shown to be potent and selective inhibitors of HIV-1 replication in culture. For example, Galactan sulfate from *Agardhiella tenera* (*Gigartinales*, *Areschongiaceae*) [8] was examined for its inhibitory effect on the cytopathic effect of HIV-1 (HTLVIIIB) and HIV-2 (LAV-2ROD), the compounds were found to provide protection at a concentration 10-fold higher than the concentration at which dextran sulfate (Mw 5 kDa) protected the cells (50% inhibitory concentration IC_50_ approximately 0.5 and 0.05 μg/mL, respectively). Witvrouw *et al.* [61] demonstrated that galactan sulfated and dextran sulfate block the binding of HIV-1 to the MT-4 cells (IC_50_ approximately 40.0 μg/mL and <0.20 μg/mL, respectively). Bourgougnon *et al*. [18] described the annual variation in composition and *in vitro* anti-HIV-1 activity of the sulfated glucuronogalactan from *Schizymenia dubyi*. Evaluation of the anti-HIV-1 effect of this polysaccharide indicated that *syncitia* formation and HIV-associated reverse transcriptase *in vitro* were completely suppressed at 5 μg/mL by the glucuronogalactan extracted from algae collected during the spring/summer period. The *syncitia* formation was completely suppressed with 5 μg/mL of galactan extracted from *S. dubyi* collected from April to July. This high anti-HIV-1 activity corresponded with a decrease in l-galactose, viscosity and sulfate content of the polysaccharide.

##### 2.4.2.1. First Approach to Determining the Mode of Action

The time of action of the polysaccharide from *S. coronopifolius* was studied by adding the compound at different times of the experiment (Table 5).

Incubation before and during (a), or presence of sulfated polysaccharide from *S. coronopifolius* only during infection (b) with cells, was ineffective to block viral infection and the viral production was comparable with virus control (Table 5). To achieve its optimal effect on *syncitium* formation, the polysaccharide must be present during the whole culture (treatment d) or after infection (treatment c).

The time of action of the polysaccharide from *B. thuyoides* was studied by adding the compound at different times of the experiment (Table 6).

Incubation before and during (a) or presence of sulfated polysaccharide from *B. thuyoides* during infection (b) with cells was ineffective to block viral infection and the viral production was comparable with virus control (Table 6). To achieve its optimal effect on syncitium formation, the polysaccharide must be present during all the culture (treatment d) or after infection (treatment c).

##### 2.4.2.2. Virus Neutralization Assay

To check the direct action of the products on the virus, they were incubated directly with the virus before adding CEM (Human T-Lymphoblastoid) cells. Even on cells CEM, the sulfated polysaccharide of *S. coronopifolius* is active at 5 μg/mL (100% of neutralization on the viral dilution of 5 × 10^−6^). The sulfated polysaccharide of *B. thuyoides* showed 42% of neutralization on highest amount of virus (7.5 × 10^−6^) but 100% of neutralization on the 5 × 10^−6^ viral dilution. These results suggest that polysaccharide had a virucidal effect.

Activity of the polysaccharide after infection demonstrates that the compound blocks the replication of HIV-1 and then the *syncitium* formation between uninfected and infected cells. Polysaccharides are active when they are in contact only after the infection or throughout, which is not due to entry of the virus into the cells, but on the viral replication itself (like witness AZT, inhibitor of the reverse transcriptase). These results suggest a direct inhibitory effect on HIV-1 replication by controlling the appearance of the new generations of virus and potential virucidal effect. The mechanism of anti-HIV action is generally attributed to the inhibition of virus attachment to the cell surface by ionic interactions between polysaccharides and cells. Preincubation of sulfated polysaccharides with cell rather than virus has generally been shown to be necessary for effective inhibition [62]. In this study, the polysaccharide of *S. coronopifolius* and *B. thuyoides*, also shows inhibition when the polysaccharide is added after infection. Direct antiviral action of sulfated polysaccharides has, however, also been described and confirms our findings [6,7,18]. Furthermore, antiviral activity increases with increasing molecular weight and degree of sulfation [18,35]. The polysaccharide of *S. coronopifolius* contains more galactose, uronic acid and sulfates than polysaccharide of *B. thuyoides*. However, the polysaccharide from *S. coronopifolius* presents a slightly lower molecular weight but is more homogeneous than polysaccharide of *B. thuyoides*.

Research on effective chemotherapeutic treatment against HIV infection led to the development of agents which aim at specific and critical events in the replicative cycle of HIV. The conjugates of κ-carrageenans and AZT (3′-azido-3′-deoxythymidine) exert synergistic effects on the reduction of the infection of the human immunodeficiency virus (HIV) [63].

In conclusion, marine polysaccharides prevent *syncytium* formation, and thus protect uninfected cells from being killed by the HIV-infected cells. With inexpensive, water-soluble and poorly absorbed properties, they are also endowed with activity against various viruses other than HIV, including viruses such as HSV and CMV that occur as opportunistic pathogens in immuno-suppressed (*i.e*., AIDS) patients. Sulfated polysaccharides constitute actually attractive candidate for a vaginal anti-HIV formulation. They can be prepared and made available in large quantities at reasonable cost.

## 3. Experimental Section

### 3.1. Extraction Techniques and Chemical Analysis Methods

*Water extraction*: Samples of *S. coronopifolius* and *B. thuyoides* were collected in 2007 at Belyounech (Strait of Detroit Gibraltar, Morocco). The samples were rinsed with sterile seawater to remove associated debris and necrotic parts. Epiphytes were removed from the algae. The samples were shade dried, cut into small pieces and powdered in a mixer grinder. The powder obtained was preserved cold (−12 °C). The seaweed was depigmented with absolute ethanol and acetone. Polysaccharides from seaweed powder (20 g) were extracted in hot distilled water (1.5 L) at 80 °C for 4 h with magnetic stirring. Insoluble residues were eliminated by filtration and centrifugation (20 min, 30,000 g). The supernatant was poured into 2 volumes of absolute ethanol during one night at 4 °C. The precipitate were recovered and washed by absolute acetone, dried overnight at 50 °C, weighed and ground to a powder. The polysaccharide was redissolved in distilled water, dialyzed against distilled water and freeze-dried [26,57].

### 3.2. Chemical Composition

Total carbohydrate was estimated by the phenol-sulfuric acid method of Dubois [64], using galactose as standard. Uronic acids were determined by m-hydroxydiphenyl of method from Blumenkrantz and Asobe-Hansen [65], modified by Filisetti-Cozzi and Carpita [66], using glucuronic acid as standard. For the determination of sugar composition, the monosaccharide residues released by acid hydrolysis were converted into their alditol acetate [67,68] and analyzed by GLC. The 3,6-anhydrogalactose content (3,6-AG) was determined by the colorimetric method of Yaphe and Arsenault [69]. Sulphate content was measured turbedimetrically after hydrolyzing 20 mg of polysaccharide in a sealed tube with 2N HCl at 100 °C for 2 h [70]. Protein content was estimated according to the method Bicinchoninic Acid (BCA) Protein Assay [71]. Pyruvic acid was determined by the 2,4-dinitrophenylhydrazine colorimetric method of Sloneker and Orentas [72] after hydrolysis (1 N chlorydric acid, 100 °C, 3 h).

### 3.3. Molecular Mass

The system is composed of an HPLC system Prominence Shimadzu™, a PL aquagel-0H mixte, 8 μm (Varian Palo Alto, CA, USA) guard column (U7.5 mm × L50 mm), and a PL aquagel-0H mixte (Varian) separation column (U7.5 mm × 300 mm, operating range 10^2^–10^7^ g/mol). Elution was performed at 1 mL/min with 0.1 M ammonium acetate containing 0.03% NaN_3_, filtrated on 0.1 μm membrane (Durapore Membrane, PVDF, Hydrophilic type VVLP, Millipore). Samples were filtrated on 0.45 μm cellulose acetate syringe filter prior to injection (100 μL). A differential refractive index (RI) detector (Hitachi L2490) and a multi-angle light scattering detector (mini-Dawn™ Treos™, Wyatt) were coupled on-line and data computed with Astra software for absolute molar mass determination [73].

### 3.4. FT-IR Spectroscopy

Infrared spectra were recorded from a KBr pellet of the polysaccharide on a spectrometer IR-TF Nicolet.

### 3.5. Determination of Antiviral Activity of Sulfated Polysaccharides

#### 3.5.1. Cells and Viruses

African green monkey kidney cells (Vero, ATCC CCL-81) were grown in Eagle’s minimum essential medium (MEM, Eurobio) supplemented with 8% fetal calf serum (FCS, Eurobio) and 1% of antibiotics PCS (10,000 IU/mL penicillin, 25,000 IU/mL colimycin, 10 mg/mL streptomycin; Sigma). HSV-1 (wild type strain 17, sensitive to acyclovir) was obtained from Pr. Billaudel (Laboratory of Virology of Nantes, France).

MT4 and CEM (human lymphocyte cell) cell lines are human CD4^+^ lymphocytes transformed by HTLV-1. They were grown in RPMI 1640 medium containing 10% foetal calf serum (FCS) (Dutscher), 1% penicillin-neomycin-streptomycin antibiotics (PNS) (Whittaker), 1% glutamine (Whittaker), 2 μg/mL polybrene (Whittaker) and re-fed twice a week. The HIV-1 NDK strain was prepared from the supernatant of an infected CEM cell line.

#### 3.5.2. Cytotoxicity Assays Based Upon Cell Viability

Using the Vero cell/HSV-1 model, cytotoxicity was evaluated by incubating cellular suspensions (3.5 × 10^5^ cells/mL) with various dilutions (concentration from 2.5 to 250 μg/mL, 4 wells per concentration) of polysaccharide in 96-well plates (48 h, 37 °C, 5% CO_2_) in Eagle’s MEM containing 8% FCS. The cells were examined daily under a phase-contrast microscope to determine the minimum concentration of polysaccharide that induced alterations in cell morphology, including swelling, shrinkage, granularity and detachment. Cytotoxicity by cell viability was tested using the neutral red dye method from Le Contel [74]. Optical density (OD) was measured at 540 nm using a spectrophotometer (SpectraCountTM, France, Packard). The 50% cytotoxic concentration (CC_50_) was defined as the concentration that reduced the OD of treated cells to 50% of that of untreated cells.

CC_50_ values were expressed as the percentage of destruction (%*D*): [(ODc)C − (ODc)MOCK/(ODc)C] × 100. (ODc)C and (ODc)MOCK were the OD values of the untreated cells and treated cells, respectively [75].

Using the MT4/HIV model, cytotoxicity was evaluated by incubating MT4 cells (3 × 10^5^ cells/mL) with the polysaccharides (final concentration 100–300 μg/mL) in 96-well plates during 1 h at 37 °C with 5% CO_2_. The MT4 cells, which are highly susceptible to the HIV cytopathic effect, were then cultured at 3 × 10^5^ cells/mL in 24-well microtiter plates containing various dilutions of polysaccharides. After 4 days, the cell viability was measured by the dead cells exclusion with trypan blue. The 50% cytotoxic concentration (CC_50_) was defined as the dose of the compound causing 50% cell death after 96 h of incubation at 37 °C [75].

#### 3.5.3. Antiviral Assays Based Upon Cell Viability

Using the Vero cell/HSV-1 model, 100 μL of cellular suspension (3.5 × 10^5^ cells/mL) in Eagle’s MEM containing 8% FCS were incubated with 50 μL of a dilution of filtered polysaccharide (concentration from 2.5 to 250 μg/mL) in 96 well-plates (48 h, 37 °C, 5% CO_2_). Three replicates were infected using 50 μL of medium and a virus suspension at a MOI of 0.001 ID_50_/cells. After incubation, antiviral activity was evaluated by the neutral red dye method. The antiherpetic compound acyclovir [9-(2-hydroxyethoxymethyl) guanine] was used as reference inhibitor. The 50% effective antiviral concentration (EC_50_) was expressed as the concentration that achieved 50% protection of virus-infected cells from virus-induced destruction. The OD was related directly to the percentage of viable cells, which was inversely related to the cytopathic effect (CPE). The linear regression was determined for each assay on the basis of cell controls (0% CPE) and virus controls (100% CPE). Data were expressed as a percentage of protection (%*P*): [((ODt) virus − (ODc) virus)/((ODc)MOCK − (ODc) virus)] × 100. (ODt) virus was the OD of the test sample, (ODc) virus was the OD of the virus control, and (ODc)MOCK was the OD of the mock-infected control [75].

Using the cells MT4/HIV model, MT4 cells were first pre-incubated during 1 h at 37 °C in 96-well microtiter plates in RPMI 1640 culture medium with 10% FCS and 1% PNS antibiotics, 1% glutamine and 2 μg/mL polybrene and containing various concentrations of polysaccharide (3 × 10^5^ cells/mL for 100 μL of compound). Infection of MT4 cells was performed by adding into wells 100 μL of HIV-1 suspension (dilution 10^−3^). One hour after incubation at 37 °C, infected cells were washed three times with RPMI and cultured at 3 × 10^5^ cells/mL in 24-well microtiter plates in the presence of polysaccharide. Cultures were grown for 7 days at 37 °C, under 5% CO_2_ atmosphere and re-fed at day 3 post-infection with cell culture medium. After 4 days, the appearance of *syncitium* was followed every day on an inverted optical microscope. Controls were HIV-1-infected MT4 cells cultured without polysaccharide (control MT4/HIV-1) and uninfected MT4 cells (control MT4). Positive control was HIV-1 infected MT4 cells cultured with a dose of 0.1 μM AZT known to give 100% inhibition of virus replication (control AZT). The virus dilution used in the assay allowed syncitium formation at day 4 post-infection. The 50% inhibitory concentration (IC_50_) of the compounds was expressed as the dose that caused 50% inhibition of viral production without direct toxicity to cells at day 7 post-infection. The therapeutic index (T1) was defined as the ratio of the 50% cytotoxic concentration to the 50% inhibitory concentration (CC_50_/IC_50_) [6].

#### 3.5.4. Virus Neutralization Assay

In order to confirm the direct action or not on the virus, a test of neutralization was carried out on CEM cells. In this protocol, the products are put in contact firstly with the virus, and then CEM cells are added. HIV-1 suspension (50 μL) were pre-incubated during 1 h at 37 °C in 96-well microtiter plates in RPMI 1640 culture medium with 10% FCS and 1% PNS antibiotics, 1% glutamine and 2 μg/mL polybrene and containing various concentrations of polysaccharides (50 μL). Then, CEM cells are added (5 × 10^5^ cells for 100 μL of virus-compound mixture). One hour after incubation at 37 °C, three washings are carried out then the pellets are resuspended in medium RPMI and transferred in a 24 well microtiter plate in 1 mL of polysaccharide or medium RPMI for the controls cells and virus. The plates are placed at 37 °C and 5% of CO_2_. From day 3 or 4, the activity of the reverse transcriptase is measured every 3 days; the cells are re-fed with new medium and the cellular concentration adjusted to 5 × 10^5^ cellules/mL. To measure the reverse transcriptase activity, 1 mL samples of cell-free supernatant collected at day 3, 6, and 10 post-infection were centrifuged (95,000 rpm, 4 °C, 5 min). The viral pellet was resuspended in 10 μL of NTE buffer containing 0.1% Triton X-100. The enzymatic reaction was performed in 40 μL of a reaction mixture of composition: Tris 50 mM, pH 7.8, MgCl_2_ 20 mM, KCl 20 mM, dithiothreitol 2 mM, oligo (dT) 12–18 0.25 OD/mL, poly(rA) 0.25 OD/mL and ^3^HdTTP 2.5 μCi. After 1 h at 37 °C, the reaction products were precipitated with 20% trichloroacetic acid, filtered on Millipore 0.45 μm and the β radioactivity was measured. Controls were HIV-1-infected CEM cells cultured without polysaccharide and uninfected CEM cells. Positive inhibitory control was HIV-1 infected CEM cells cultured with AZT [6].

#### 3.5.5. First Approach of Mode of Antiviral Action

A first approach to determine the mode of action polysaccharides was initiated by carrying out different treatments in the two models:

##### 3.5.5.1. HSV/Vero Model [17,53,57,76]

###### Effect before Infection

To determine whether a cellular antiviral state could be induced by polysaccharide, cells were incubated with polysaccharide (24 h, 37 °C, 5% CO_2_) and were then washed with PBS. Cells were inoculated with virus and incubated for 48 h (37 °C, 5% CO_2_). The effect on virus multiplication was determined by the neutral red dye method for HSV-1.

###### Virucidal Assay

A virus suspension containing 0.001 ID_50_/cell of HSV-1 was incubated with an equal volume of medium with or without polysaccharide dilutions (10–250 μg/mL) for 2 h at 37 °C for HSV-1. One hundred microliters of mixed suspension was then added to 100 μL of cellular suspension (3 × 10^5^ Vero cells/mL) in culture medium. After incubation for 48 h, the virucidal effect was determined using the neutral red dye method for HSV-1.

###### Virus Adsorption Assay

The inhibitory effect of polysaccharide on virus adsorption was measured on confluent monolayers of Vero cells infected with HSV-1 at 0.001 ID_50_/cell, under different treatments. In Treatment I, cells were exposed to HSV-1 in the presence of various polysaccharide dilutions. After virus adsorption (1 h at 4 °C), cells were washed with PBS to remove both polysaccharide and unadsorbed virus and further incubated with medium. In Treatment II, cells were exposed to HSV-1 and after a virus adsorption period (1 h at 4 °C), unadsorbed virus was removed and cells were further incubated with the medium containing different concentrations of polysaccharide. In Treatment III, polysaccharide was present both during and after the adsorption period. The effect on HSV-1 adsorption was determined after 2 days by the neutral red dye method.

###### Effect of Polysaccharide Addition Time

Monolayers of Vero cells were inoculated with HSV-1 at 0.001 ID_50_/cell, and polysaccharide was added simultaneously or after 1, 2, 3 or 5 h following infection. After 48 h of incubation, effect on HSV-1 replication was determined by the neutral red dye method.

##### 3.5.5.2. HIV/MT4 Model [7]

###### Before and during Infection

The concentrations range shown was in between 12.5 μg/mL to 100 μg/mL. Cells were pre-incubated for 2 h on a 96-well microtiter plate in culture medium containing different concentration of polysaccharide. Cells were infected adding into wells 100 μL of HIV-1 suspension (dilution 7.5 × 10^−5^ or 10^−4^). One hour after incubation, infected cells were washed three times with RPMI, infected and cultured without the compound.

###### During Infection

The polysaccharide was added simultaneously with the virus, and after 1 h of incubation, infected cells were washed three times with RPMI and cultured without polysaccharide.

###### After Infection

The polysaccharide was present only after infection.

###### Polysaccharide Present during All the Culture

Cells were pre-incubated for 2 h on a 96-well microtiter plate in culture medium containing different concentration of polysaccharide. Cells were infected adding into wells 100 μL of HIV-1 suspension (dilution 7.5 × 10^−5^ or 10^−4^). One hour after incubation, infected cells were washed three times with RPMI and cultured at 3 × 10^5^ cells/mL in a 24-well microtiter plate in the presence of polysaccharide.

## 4. Conclusions

Analysis of chemical compositions, evaluation of molecular mass and analysis of FT IF spectra suggested that the polysaccharide extracted *S. coronopifolius* could belong to the family of λ-carrageenans with presence of uronic acids and the polysaccharide from *B. thuyoides* to the agar family.

The sulfated polysaccharides from *S. coronopifolius* and *B. thuyoides* inhibit HSV-1 and HIV-1 replication *in vitro* at concentrations that have no effect on cell viability. The adsorption step of HSV-1 to the host cell seems to be the specific target of polysaccharide action. While for HIV-1, these results suggest a direct inhibitory effect on HIV-1 replication by controlling the appearance of the new generations of virus and having potential virucidal effect. Further studies will be necessary to establish structure-activity relationships.

## Figures and Tables

**Table 1:** Chemical composition (in % of dry weight) of the sulfated polysaccharide from *S. coronopifolius* and *B. thuyoides*.

Water-extracted polysaccharides		
	*S. coronopifolius*	*B. thuyoides*

Neutral sugars		
Galactose	33.1	25.4
Xylose	1.80	2.80
Glucose	1.70	3.00
Rhamnose	0	0.30
3,6-anhydrogalactose	11.00	16.00

Glucuronic acid	6.70	0

Galacturonic acid	1.0	3.2

Sulfate	24.00	7.60

Proteins	0	6.00

Pyruvic acid	0.34	0

Ash	13.24	13.05

**Table 2:** Time of action of *S. coronopifolius* and *B. thuyoides* polysaccharides.

M		EC_50 PSC_	EC_50 PBT_	EC_50 ACV_
Effect before infection		>250	>250	>5
Virucidal assays		53.2	73.9	-
Virus adsorption assay	Treatment I	35.4	41.1	0.8
	Treatment II	26.2	20.7	0.2
	Treatment III	3.6	13.1	0.1
Effect of time of polysaccharide addition	0 h	<2.5	10.7	0.5
	1 h after infection	<2.5	22.7	1.8
	2 h after infection	2.5	32.4	2.7
	3 h after infection	>250	>250	3.2
	5 h after infection	>250	>250	4.1

An approach to determine the mode of action of polysaccharide *Sphaerococcus coronopifolius* (PSC) and polysaccharide *Boergeseniella thuyoides* (PBT) was initiated by carrying out different treatments on the HSV-1/Vero model, and the results are shown in Table 2. Addition time: PSC or PBT was added simultaneously, or 1, 2, 3 or 5 h after virus inoculation. Treatment I: Virus + cells + PSC or PBT → adsorption during 1 h, 4 °C → washing by PBS → incubation of cells during 48 h, Treatment II: Virus + cells → adsorption during 1 h, 4 °C → washing by PBS → incubation of cells + PSC or PBT, Treatment III: Virus + cells + PSC or PBT → adsorption during 1 h, 4 °C → washing by PBS → incubation of cells + PSC or PBT. Effect before infection: cells and PSC or PBT were incubated for 24 h at 37 °C before virus inoculation. Virucidal assays: virus and PBT or PSC were incubated for 2 h at 37 °C before addition to cell suspension. Evaluation was carried out in triplicate; PBS: Phosphate buffered saline.

**Table 3:** Inhibiting effect on the HIV-1 NDK strain of the polysaccharide of *S. coronopifolius* at two viral dilutions.

	Concentration	D3		D4		D5		D6		D7	
SC 1 mg/mL	100 μg/mL	-	-	-	-	-	-	-	-	-	-
	50 μg/mL	-	-	-	-	-	-	-	-	-	-
	25 μg/mL	-	-	-	-	-	-	-	-	-	-
	12.5 μg/mL	-	-	-	-	-	-	-	-	-	-
**Control HIV-1 NDK 7.5 × 10**^−^**^4^**		(+)	(+)	+	+	++	++	T	T	T	T
		(+)/+	(+)	+/++	+	++	++/T	T	T	T	T
SC 1 mg/mL	100 μg/mL	-	-	-	-	-	-	-	-	-	-
	50 μg/mL	-	-	-	-	-	-	-	-	-	-
	25 μg/mL	-	-	-	-	-	-	-	-	-	-
	12.5 μg/mL	-	-	-	-	-	-	-	-	-	-
**Control HIV-1 NDK 5 × 10**^−^**^4^**		(+)	(+)	++	++	++/T	++/T	T	T	T	T
		-	-	+	+/++	T	++/T	T	++/T	T	T
Control cells MT4		-	-	-	-	-	-	-	-	-	-

-: absence of syncitium in all the wells; (+): presence of 1 or 2 syncitium by field; + and ++: presence of many syncitium per field; T: cellular death due to the viral replication; D: Days.

**Table 4:** Inhibiting effect on the HIV-1 NDK strain of the polysaccharide of *B. thuyoides* at two viral dilutions.

	Concentration	D3		D4		D5		D6		D7	
BT 1 mg/mL	100 μg/mL	-	-	-	-	-	-	-	-	-	-
	50 μg/mL	-	-	-	(+)	-	(+)/+	-	+	-	+
	25 μg/mL	-	-	(+)	-	+	(+)	++	++	T	T
	12.5 μg/mL	-	-	(+)/+	+	++/T	++/T	T	T	T	T
**Control HIV-1 NDK 7.5 × 10**^−^**^4^**		(+)	(+)	+	+	++	++	T	T	T	T
		(+)/+	(+)	+/++	+	++	++/T	T	T	T	T
BT 1 mg/mL	100 μg/mL	-	-	-	-	-	-	-	-	-	-
	50 μg/mL	-	-	-	-	-	(+)	-	(+)/+	-	+
	25 μg/mL	-	-	+	(+)/+	++	++	T	T	T	T
	12.5 μg/mL	(+)	-	++	+/++	++/T	++/T	T	T	T	T
**Control HIV-1 NDK 5 × 10**^−^**^4^**		(+)	(+)	++	++	++/T	++/T	T	T	T	T
		-	-	+	+/++	T	++/T	T	++/T	T	T
Control cells MT4		-	-	-	-	-	-	-	-	-	-

-: absence of *syncitium* in all the well; (+): presence of 1 or 2 *syncitium* by field; + and ++: presence of many *syncitium* per field; T: cellular death due to the viral replication; D: Days.

**Table 5:** Time of action of the sulfated polysaccharide from S. coronopifolius: Inhibition of syncitium formation by the polysaccharide added at different times of the culture: Throughout the whole experiment (Treatment (d)); before and during infection (Treatment (a)); during infection (Treatment (b)); or after infection (Treatment (c)) of MT4 cells with HIV.

Time of action		Concentration	D3		D4		D5		D6	
Before and during (a)	**SC**	12.5 μg/mL	(+)	(+)	++	++	T	T	T	T
	**AZT**	0.4 μM	(+)	(+)	+	+	T	T	T	T
During (b)	**SC**	12.5 μg/mL	+	+	+/++	+/++	T	T	T	T
	**AZT**	0.4 μM	(+)	(+)	+/++	+/++	T	T	T	T
After (c)	**SC**	12.5 μg/mL	-	-	-	-	-	-	-	-
	**AZT**	0.4 μM	-	-	-	-	-	-	-	-
All the time (d)	**SC**	12.5 μg/mL	-	-	-	-	-	-	-	-
	**AZT**	0.4 μM	-	-	-	-	-	-	-	-
**Control HIV-1 NDK 1 × 10**^−^**^4^**			++	++	++	++/++T	T	T	T	T
			++	+	++	++	T	T	T	T
Before and during (a)	**SC**	12.5 μg/mL	(+)/+	+	+/++	++	T	T	T	T
	**AZT**	0.4 μg/mL	(+)	-	+	(+)/+	++T	++T	T	T
During (b)	**SC**	12.5 μg/mL	+	+	++	++	T	T	T	T
	**AZT**	0.4 μg/mL	(+)	(+)	+	+/++	T	++T	T	T
After (c)	**SC**	12.5 μg/mL	-	-	-	-	-	-	-	-
	**AZT**	0.4 μg/mL	-	-	-	-	-	-	-	-
All the time (d)	**SC**	12.5 μg/mL	-	-	-	-	-	-	-	-
	**AZT**	0.4 μg/mL	-	-	-	-	-	-	-	-
**Control HIV-1 NDK 7.5 ×10**^−^**^5^**			+	+	+	+	T	T	T	T
			++	++	+	+/++	T	T	T	T
MT4			-	-	-	-	-	-	-	-

-: absence of *syncitium* in all the wells; (+): presence of 1 or 2 *syncitium* by field; + and ++: presence of many *syncitium* per field; T: cellular death due to the viral replication; D: days.

**Table 6:** Time of action of the sulfated polysaccharide from *B. thuyoides*: Inhibition of syncitium formation by the polysaccharide added at different times of the culture: Throughout the whole experiment (Treatment (d)); before and during infection (Treatment (a)); during infection (Treatment (b)); or after infection (Treatment (c)) of MT4 cells with HIV.

Time of action		Concentration	D3		D4		D5		D6	
Before and during (a)	**BT**	75 μg/mL	(+)	(+)	++	++	T	T	T	T
	**AZT**	0.4 μM	(+)	(+)	+	+	T	T	T	T
During (b)	**BT**	75 μg/mL	(+)	+	+/++	+/++	T	T	T	T
	**AZT**	0.4 μM	(+)	(+)	+/++	+/++	T	T	T	T
After (c)	**BT**	75 μg/mL	-	-	-	-	-	-	-	-
	**AZT**	0.4 μM	-	-	-	-	-	-	-	-
All the time (d)	**BT**	75 μg/mL	-	-	-	-	-	+	-	+
	**AZT**	0.4 μM	-	-	-	-	-	-	-	-
**Control HIV-1 NDK 1 × 10**^−^**^4^**			++	++	++	++/++T	T	T	T	T
			++	+	++	++	T	T	T	T
Before and during (a)	**BT**	75 μg/mL	+	(+)/+	++	++	++T	T	T	T
	**AZT**	0.4 μg/mL	(+)	-	+	(+)/+	++T	++T	T	T
During (b)	**BT**	75 μg/mL	+	+	++	++	T	T	T	T
	**AZT**	0.4 μg/mL	(+)	(+)	+	+/++	T	++T	T	T
After (c)	**BT**	75 μg/mL	-	-	-	-	-	-	-	-
	**AZT**	0.4 μg/mL	-	-	-	-	-	-	-	-
All the time (d)	**BT**	75 μg/mL	(+)	-	-	+	-	+	-	+
	**AZT**	0.4 μg/mL	-	-	-	-	-	-	-	-
**Control HIV-1 NDK 7.5 × 10**^−^**^5^**			+	+	+	+	T	T	T	T
			++	++	+	+/++	T	T	T	T
MT4			-	-	-	-	-	-	-	-

-: absence of syncitium in all the well; (+): presence of 1 or 2 syncitium by field; + and ++: presence of many syncitium per field; T: cellular death due to the viral replication; D: days.
